# Supplementary material for: TE-aware state analysis and evolution in dynamic network connectivity
Source: bioRxiv. 2026 Sep 25:2026.09.20.753026. Preprint. [Version 1] doi: 10.64898/2026.09.20.753026 (PMC13622153; doi:10.64898/2026.09.20.753026)
Supplement: Supplementary file 1 [file media-1.docx]

**Supplementary Information**
